# Synthesis and Cytotoxicity Studies on Ru and Rh Nanoparticles as Potential X-Ray Fluorescence Computed Tomography (XFCT) Contrast Agents

**DOI:** 10.3390/nano10020310

**Published:** 2020-02-12

**Authors:** Yuyang Li, Kian Shaker, Martin Svenda, Carmen Vogt, Hans M. Hertz, Muhammet S. Toprak

**Affiliations:** Biomedical and X-Ray Physics, Department of Applied Physics, KTH Royal Institute of Technology, SE 10691 Stockholm, Sweden; yuyangli@kth.se (Y.L.); kiansd@kth.se (K.S.); martin.svenda@biox.kth.se (M.S.); carmenma@kth.se (C.V.); hans.hertz@biox.kth.se (H.M.H.)

**Keywords:** polyol synthesis, nanoparticles, bio-imaging, X-Ray fluorescence, contrast agent, XFCT

## Abstract

X-Ray fluorescence computed tomography (XFCT) is an emerging biomedical imaging technique, which demands the development of new contrast agents. Ruthenium (Ru) and rhodium (Rh) have spectrally attractive K_α_ edge energies, qualifying them as new XFCT bio-imaging probes. Metallic Ru and Rh nanoparticles are synthesized by polyol method, in the presence of a stabilizer. The effect of several reaction parameters, including reaction temperature time, precursor and stabilizer concentration, and stabilizer molecular weight, on the size of particles, were studied. Resultant materials were characterized in detail using XRD, TEM, FT-IR, DLS-zeta potential and TGA techniques. Ru particles in the size range of 1–3 nm, and Rh particles of 6–9 nm were obtained. At physiological pH, both material systems showed agglomeration into larger assemblies ranging from 12–104 nm for Ru and 25–50 nm for Rh. Cytotoxicity of the nanoparticles (NPs) was evaluated on macrophages and ovarian cancer cells, showing minimal toxicity in doses up to 50 μg/mL. XFCT performance was evaluated on a small-animal-sized phantom model, demonstrating the possibility of quantitative evaluation of the measured dose with an expected linear response. This work provides a detailed route for the synthesis, size control and characterization of two materials systems as viable contrast agents for XFCT bio-imaging.

## 1. Introduction

X-Ray fluorescence (XRF) computed tomography (XFCT) is an emerging modality of biomedical imaging. By detecting the characteristic Kα X-rays emitted from the contrast agents, in vivo spatial distribution and elemental analysis can be achieved under post-imaging reconstruction. With the laboratory XFCT setup Larsson et al. reported the 3D imaging of MoO_2_ nanoparticles (NPs) in rodents with 200 μm spatial resolution at acceptable X-Ray radiation dose and exposure time with XFCT setup [1,2]. This extends the application of X-Ray CT imaging from structural imaging to molecular imaging Clinically used molecular imaging modalities include magnetic resonance imaging (MRI), optical fluorescence, ultrasound, positron emission tomography (PET) and single-photon emission computed tomography (SPECT) [3]. These imaging techniques all require the use of specific contrast agents. For example, superparamagnetic iron oxides as the magnetic nanomaterials for MRI imaging; quantum dots gold and rare earth oxide NPs for optical imaging; silica NPs for ultrasound imaging; radionuclide-labeled compounds for nuclear imaging (PET, SPECT). Likewise, a library of NPs based on yttrium (Y), zirconium (Zr), niobium (Nb), molybdenum (Mo), ruthenium (Ru), and rhodium (Rh) elements has shown them as promising XFCT contrast agent platforms owing to their spectrally matching Kα edge energies to the liquid–metal–jet X-Ray source [4].

Ru and Rh NPs have received much attention mainly for their application as catalysts in hydrogenation, ethanol steam reforming and CO oxidation, as well as for their use in electronics and optics [5]. In addition, some Ru and Rh organometallic complexes have been used in biological applications for their luminescence properties [6]. A series of Ru complexes have been studied in recent years as anticancer agents for the specific feature of interaction towards DNA targeting, showing high anticancer activity both in vitro and in vivo; therefore, parts of Ru-based anticancer drugs are under clinical investigation [7,8,9,10]. There is/are very limited research/data on the biocompatibility and biomedical use of metallic Ru and Rh NPs, unlike other noble metals such as gold, silver, and platinum. Only in a recent study on cancer phototherapy, were metallic Rh nanostructures (nanoshells, nanoframes and porous nanoplates, with size around 100 nm) reported with promising biocompatibility [11].

For the synthesis of metallic NPs, there are several methods including chemical reduction [12], UV photolysis, thermal decomposition [13], metal vapor deposition [14], electrochemical reduction [15] sonochemical decomposition [16], and microwave irradiation [17]. Among these, the chemical reduction method is a rapid and easily scalable route to prepare water-dispersible Ru and Rh NPs. The process can be done in an aqueous solution using reducing agents like NaBH_4_, or in long-chain alcohol media (also known as polyol synthesis), where the alcohol acts as the reducing agent and solvent at the same time.

In this work, we report on the synthesis of size-controlled metallic Ru and Rh NPs by the chemical reduction method using polyol synthesis. By adjusting the amount of precursors and the stabilizer (PVP), the reaction temperature and time, and the amount and molecular weight of the stabilizer, Ru and Rh NPs with different size and extent of agglomeration were obtained. The structural and physicochemical characteristics, and the cytotoxicity of these materials were studied, finally demonstrating their applicability as contrast agents for XFCT.

## 2. Materials and Methods

### 2.1. Materials

Rhodium (III) chloride hydrate (RhCl_3_·xH_2_O, Rh 38.5%–45.5%), Ruthenium (III) chloride hydrate (RuCl_3_·xH_2_O, Ru 38%–40%), Ethylene glycol (>99%), and Poly (vinyl pyrrolidone) (PVP, average MW = 55 kDa, 10 kDa) were obtained from Sigma Aldrich. Hydrochloric acid, Sodium hydroxide and solvents including acetone, ethanol of analytical grade were obtained from Sigma Aldrich. All chemicals were used without further purification.

### 2.2. Synthesis of Rh and Ru NPs

The synthesis process was a polyol reduction using ethylene glycol (EG) as the solvent. Specifically, a Ru or Rh precursor (0.2 mmol of Rh/Ru atom) and PVP as the stabilizer (4 mmol in repeating units) were dissolved in 20 mL EG using a glycerol bath. The solution was stirred vigorously in a 50 mL three-neck flask fitted with a condenser connected with cooling tap water. During the synthesis, the temperature of the glycerol bath was controlled using a thermocouple and the reaction temperature was monitored with a thermometer. Rh solution was heated to a nucleation temperature of around 85 °C, where particle nucleation began. This was macroscopically observed as a darkening in the solution’s color. Due to the much slower kinetics of Ru nucleation, no clear nucleation temperature up to 140 °C was observed. After 15 min, the Rh/Ru solution was heated to the focusing temperature, which was observed as a further darkening in the reaction mixture. The reaction was kept for 1.5 h at the focusing temperature before being quenched. NPs were washed three times by successive precipitation in acetone, then centrifuged and re-dispersed in DI water. The obtained NPs were stored in DI water for further analyses. A series of reactions were performed for tuning size of NPs, by adjusting the amount of precursors and stabilizer (PVP), and the reaction temperature-time, amount and molecular weight of the stabilizer. Details of all synthesized samples are given in Table 1.

### 2.3. Characterization Methods

The crystal structure and crystallinity of as-prepared materials in powder form, after drying the collected particles in a vacuum oven overnight, were investigated by X-Ray powder diffraction (PANalytical Xpert Pro alpha powder, PANalytical) with Cu Kα radiation (λ = 1.54056 Å). The dry particle size, morphology, and crystallinity of the samples were studied using transmission electron microscopy (TEM) (JEM-2100F, 200 kV, JEOL Ltd., Japan). The samples were prepared by drop casting ~20 µL of colloidal suspension on a TEM grid and allowing them to dry overnight. From the TEM micrographs, primary particle size was measured on at least 200 NPs in different fields of view. Fourier-transform infrared spectroscopy (FT-IR, Thermo Scientific Nicolet iS20, Stockholm, Sweden) was used to obtain FT-IR spectra in transmission mode (KBr Mini-Pellet Press, Specac, Sigmaaldrich, Stockholm, Sweden) in the 4000–400 cm^−1^ range. Dynamic light scattering (DLS, Malvern Nano-ZS90) was used to investigate the hydrodynamic size distribution of the as-prepared particles dispersed in DI water, adjusted to pH 7.5. Using the same system, the surface charge, i.e., zeta potential, of as-synthesized NPs has been evaluated as a function of pH. Thermogravimetric analysis (TGA 550, TA instruments, Sollentuna, Sweden) was used to study the composition of the dried nanomaterials samples. Inductively coupled plasma–optical emission spectroscopy (ICP-OES) (iCAP 6000 series, Thermo Scientific) was used for the determination of the elemental composition of the as-synthesized materials prior to XFCT phantom experiments.

### 2.4. In Vitro Toxicity

Toxicity tests were performed on two cell lines; murine macrophages (RAW 264.7, ATTC TIB-71, Sigmaaldrich, Stockholm, Sweden) and human-derived ovarian cancer (SKOV-3, ATCC HTB-77, Wesel, Germany) cell lines using Cell Counting Kit-8 (CCK-8) (Cat. # 96922, Sigma Aldrich, Stockholm, Sweden).

Dulbecco’s modified Eagle medium (DMEM) containing 10% fetal bovine serum (FBS) was used as the cell media. As negative control, cells were grown in the same media as above in the absence of NPs. The cells were about 80% confluent when the assay was performed.

The cells were split and seeded into the wells of 96-well plates (Cat. # 167008, Thermo Fisher, Stockholm, Sweden) 24 h before start of NP exposure. Twenty-four hours after seeding, the cells were exposed for another 24 h to a dilution series of the two metallic NPs. The amount of NPs in the first well of the dilution series was a 10-fold dilution of the stock, and then a five-fold dilution series was performed to obtain a concentration series. Since the NPs were dark, which could potentially disturb absorbance measurements during the assays, the media in all the wells used were aspirated off and new fresh media were added just before the initiation of the assays.

The CCK-8 assay was performed according to the provider’s instructions and the absorbance measurements were done on a Synergy LX multi-mode reader (BioTek Instruments, Solna, Sweden). Briefly, the assay was performed by adding the substrate, 10 µL WST-8 (2-(2-methoxy-4-nitrophenyl)-3-(4-nitrophenyl)-5-(2,4-disulfophenyl)-2H-tetrazolium, monosodium salt) to 100 µL cell media. The plates were incubated at 37 °C, 5% CO_2_ for 2 h, after which the absorbance was measured. During incubation, the substrate was reduced by enzymes of active cells to form an orange formazan dye with a different absorption wavelength compared to the original substrate. The amount of formazan produced is directly proportional to the number of living cells.

### 2.5. XFCT Phantom Experiments

In order to investigate the contrast potential of the NPs in a small-animal XFCT setting, experiments were performed on a phantom of small-animal size and material. A 30 mm diameter hollow cylinder of polylactic acid (PLA) was 3D-printed and filled with water to simulate soft tissue. Six cylindrical inserts of 3 mm diameter each were filled with different concentrations of Ru (Ru-2) and Rh (Rh-2) NPs dispersed in water, yielding specifically the following concentrations: 1.0, 0.8, 0.6, 0.4, 0.2 and 0.1 mg/mL (see Figure 8).

The phantom was then scanned using our laboratory arrangement for simultaneous XFCT and computed tomography (CT). For a detailed description of the imaging arrangement, see Reference [1], and Reference [18]. Scanning was performed with 200 μm step size for each projection, of which 60 were acquired over 180 degrees for a tomographic scan. At each step, the phantom was exposed to X-rays for 10 ms, resulting in a total scan time of ~3 min for a single axial slice, for a radiation dose of ~50 mGy per slice, estimated using Monte Carlo simulations [19]. The CT data was then reconstructed using a standard filtered back projection algorithm, while the XFCT data was reconstructed using in-house developed iterative algorithms. The latter considers the self-absorption of XRF inside the phantom, meaning that the concentration of NPs can be reconstructed quantitatively.

## 3. Results and Discussion

### 3.1. Mechanism of NP Formation

Polyol synthesis is a promising route for the synthesis of various metallic NP systems, where the polyalcohol solvent also acts as the reducing agent for the metallic ions in the solution. These reactions take place at elevated temperatures to favor the thermodynamics and kinetics of the process. The mechanism of synthesis is through the reduction in Ru^3+^/Rh^3+^ ions to metallic Ru/Rh by the solvent EG- at temperatures lower than the boiling point of EG, directly forming the crystalline Ru/Rh NPs- without needing any further thermal treatment. In parallel with the reduction reaction, the solvent (EG) is oxidized to diacetyl molecule step-wise. The overall reaction taking place during the formation of Ru/Rh NPs can be schematically represented as follows, where M^0^ represents Ru or Rh NP formation in the reaction

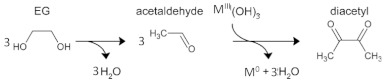



The surface chemistry of the resultant particles will be mostly determined by the organics present during the synthesis process. PVP was used here as a stabilizer molecule, forming ion (Ru^3+^/Rh^3+^) pools before the reduction process. Therefore, the amount of PVP in the reaction is an important parameter to adjust in order to tune the size of the formed metallic particles

### 3.2. Characterization of Crystallinity, and Surface Adsorbed Groups

**Figure 1:**
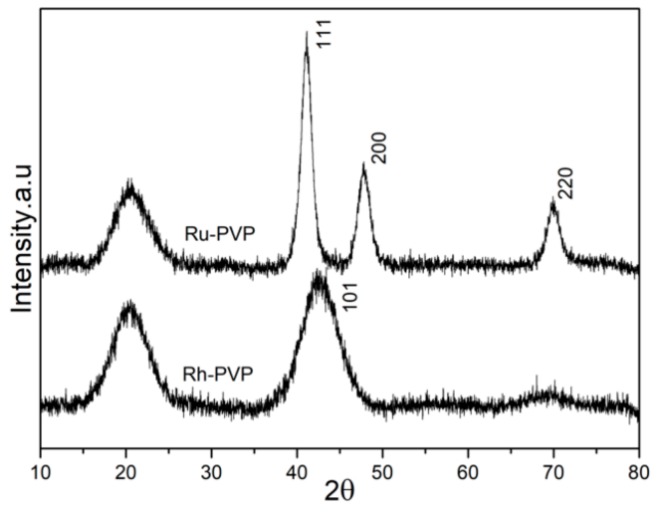
X-Ray powder diffraction patterns of as-synthesized Ru (Ru-2) and Rh (Rh-2) nanoparticles (NPs) by polyol synthesis. The Miller indices for the observed diffraction peaks are based on the structure match with the following powder diffraction files: Ru (ICDD card#: 01-089-4903), Rh (ICDD card#: 03-065-2866).

Ru and Rh NPs, that were synthesized through chemical reduction using polyol method, were dried and analyzed for their crystal structure and crystallinity. All samples have been analyzed and showed similar diffraction patterns and surface chemistry in the series of each element. One sample from each series is chosen for the cytotoxicity tests and their detailed results are presented, representing the family of samples listed in Table 1. The X-Ray powder diffraction patterns of selected samples Ru-2 and Rh-2 (see Table 1 for details) are shown in Figure 1. Both samples showed broad diffraction patterns which matched with the corresponding powder diffraction cards for Ru (ICDD card: 01-089-4903), and Rh (ICDD card: 03-065-2866). The diffraction peaks were rather broad due to the small crystallite size and non-uniform strain within the materials. Consequently, there was only very limited number of diffraction planes that were visible in the XRD patterns. The common broad peak observed at 21° in the XRD pattern of both samples was due to the presence of PVP—in agreement with the previous report on PVP-stabilized, Ag composite microspheres [20].

**Figure 2:**
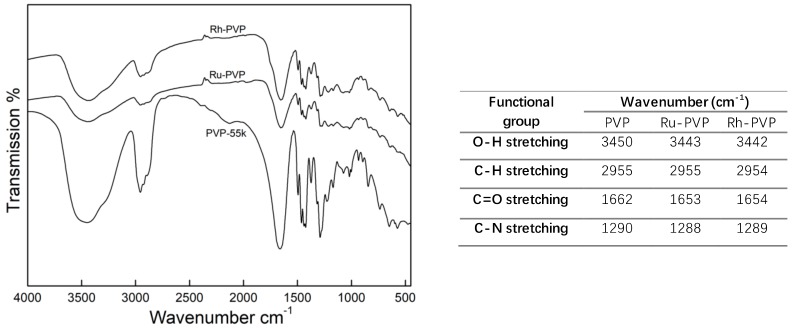
Fourier-transform infrared spectra (FT-IR), and assignment of observed bands to the functional groups, for pure PVP (55 kDa) and as-synthesized, PVP-coated Ru-2 and Rh-2 NPs.

PVP is a polar water-soluble polymer, commonly used as a stabilizer for NP surface functionalization. The PVP chains were grafted onto the NPs surface during the energetic reaction. The presence of an organic coating layer on Ru and Rh NPs is proved by the thermal gravimetric analysis (TGA; Supplementary Information Figure S1). In order to confirm the interaction between the NPs’ surface and the PVP, FT-IR analysis of Ru and Rh NPs and pure PVP (55 kDa) was performed (Figure 2). Typical absorption bands for PVP were observed in the spectra for Ru and Rh NPs, indicating the presence of PVP on their surface. Specifically, the absorption bands of C=O and CH_2_- bond stretching, at 1627 cm^−1^ and 1421 cm^−1^, and C-N bond vibration at 1287 cm^−1^, matched the absorption bands of PVP. Furthermore, the absorption band of C=O bond was shifted to shorter wavenumbers, indicating that the surface of Ru and Rh NPs was interacting with the PVP via C=O group [21].

### 3.3. Morphology, Surface Chemistry and Size Distribution Analysis of NPs

**Figure 3:**
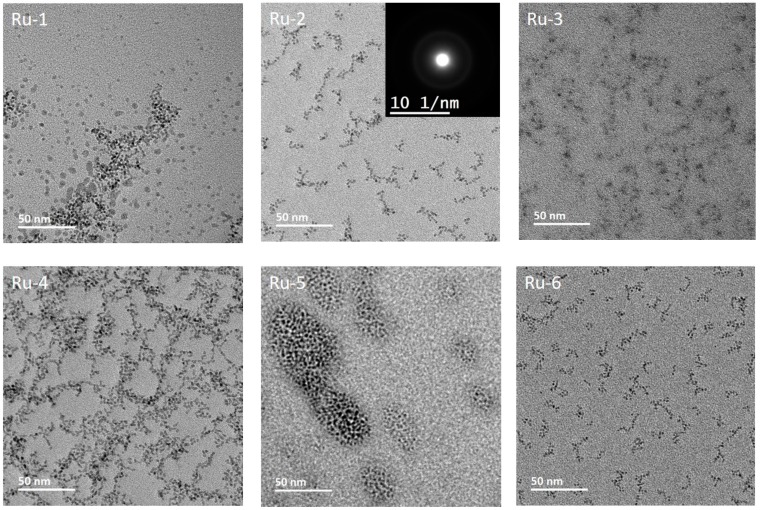
TEM micrographs of the as-synthesized Ru NPs labeled with the name of the respective samples. For the details of synthesis and labeling, see Table 1.

**Figure 4:**
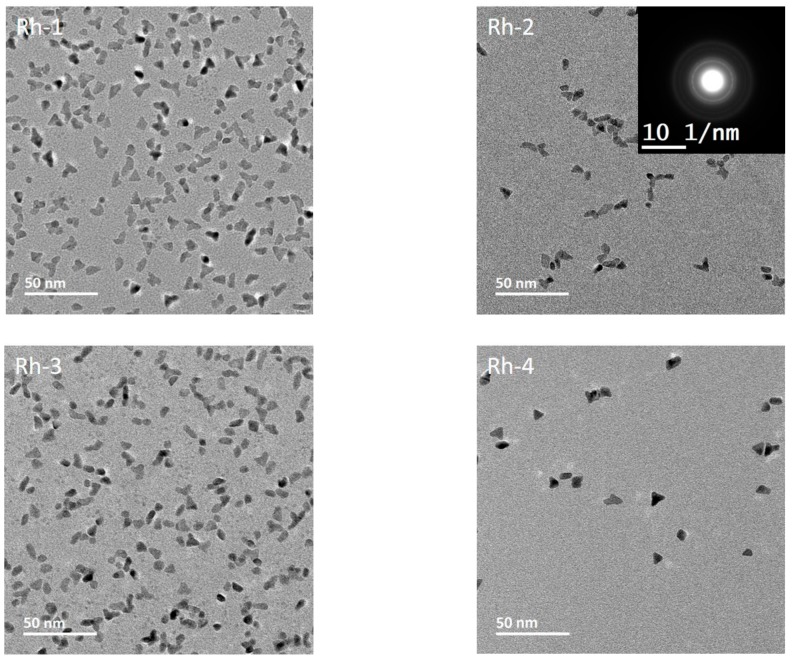
TEM micrographs of as-synthesized Rh NPs labeled with the name of respective samples. For the details of synthesis and labeling, see Table 1.

Transmission electron microscopy (TEM) micrographs of the as-prepared Ru and Rh NPs are presented in Figure 3 and Figure 4. Figure 3 shows the six samples of Ru NPs, prepared by changing the concentration and molecular weight (MW) of PVP with the reaction time, focusing temperature, and the amount of metal precursors. Table 1 summarizes the series of experiments performed to investigate the influence on various reaction parameters on the particle size of Ru and Rh. The morphology of Ru NPs was observed to be spherical/spheroidal, with a particle size in the range of 1–3 nm, while Rh displayed spheroid and triangle like particles with size in the range of 6–9 nm.

To identify the optimum temperature for the synthesis process, Ru NPs have been prepared at three different temperatures—140 °C (Ru-5), 150 °C (Ru-2) and 160 °C (Ru-1)—under otherwise identical conditions. Primary particle sizes were estimated from the TEM micrographs as 1.5, 2.5 and 1.6 nm, respectively. Reaction kinetics is improved by increasing temperature in general. At 140 °C, the kinetic of the reaction is slower, which leads to small particles despite a long reaction times and probably a high concentration of unreacted precursor in the final reaction solution. At 150 °C, particles suffer Ostwald ripening (a process of dissolution of smaller particles at the expense of forming larger ones to minimize the interfacial energy) and grow larger. At 160 °C, the nucleation kinetics will improve and burst nucleation will yield a larger number of smaller nuclei, leading to smaller particles than at 150 °C. We, therefore, have chosen 150 °C as the ideal synthesis temperature for Ru NPs to study the influence of other reaction parameters. Ru-2 and Ru-4 samples were prepared at a reaction temperature of 150 °C by varying the concentration of the PVP. Primary particle size, estimated from TEM micrographs, was about 2.5 nm for both the samples, showing no significant impact of PVP concentration for the synthesized NPs, which could be due to an excess of PVP in the reaction.

Synthesis of Ru-2 and Ru-3 were performed at 150 °C by reducing the concentration of Ru precursor by half in Ru-3 compared to Ru-2. The lower precursor concentration in Ru-3 resulted in very small particles of < 1 nm, which was an expected outcome of precursor-limited reaction kinetics. Ru-2 and Ru-6 samples were synthesized at 150 °C by reducing the reaction time to 0.5 h (Ru-6) compared to 1.5 h (Ru-2). NP size was reduced to 1.6 nm for Ru-6 at a shorter reaction time. Ostwald ripening took place minimally in short reaction periods, with the result of smaller sized NPs.

A series of Rh NPs were prepared by varying the concentration and molecular weight of PVP, as well as the reaction temperature. The corresponding TEM micrographs are presented in Figure 4. A lower reaction temperature has been identified for Rh, due to its more favorable reduction potential. The influence of PVP MW has been tested in Rh-1 and Rh-2 samples by changing the MW from 10 to 55 kDa. The primary particle size was about 15% smaller for Rh-2 (6 nm) as compared to Rh-1 (7 nm). Larger PVP chains can simply form stiffer and smaller ion pools prior to reduction, confining the size of particles formed. The effect of PVP concentration has been studied in samples Rh-2 and Rh-3, by halving the concentration in sample Rh-3 compared to sample Rh-2. The primary particle size for Rh-2 (6 nm) was about 10% smaller than that of Rh-3 (6.4 nm). Lowering the PVP content will generate slightly looser ion pools, leading to larger particles. The effect of reaction temperature was studied in Rh-2 (115 °C) and Rh-4 (150 °C), leading to primary particle sizes of 6.4 nm and 8.7 nm, respectively. A larger particle size was achieved at higher temperatures due to improved kinetics, effectively consuming precursors present in the reaction mixture.

It is important to note that Ru and Rh NP samples prepared under the same conditions of precursor concentration, reaction temperature, reaction time, and the same MW of PVP possess significantly different particle sizes, as exemplified by samples Ru-2 and Rh-4 (see Table 1 for experimental details). From the TEM micrographs, Ru-2 was observed to have an average particle size of 2.5 nm while Rh-4 has 8.7 nm. The observed difference in the size of Ru and Rh NPs prepared under the same conditions is related to their standard reduction potential, where a higher reduction potential corresponds to a larger particle size [22]. The difference in size between Ru and Rh series, therefore, can be ascribed to the lower reduction potential of Ru^3+^ (0.60 mV), as compared to that of Rh^3+^ (0.76 mV).

**Figure 5:**
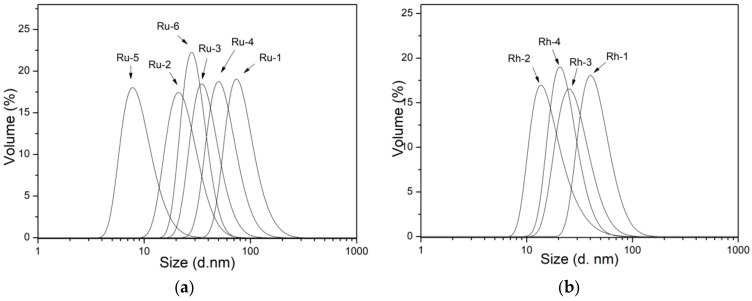
Dynamic light scattering (DLS) size distribution plots of as-synthesized (**a**) Ru and (**b**) Rh NPs dispersed in DI water at pH 7. For the details of labeling see Table 1.

It is essential to investigate the dispersed size of NPs designed in relevant media for bio-medical applications, as the dispersed size may influence the interaction with the biological systems. Therefore, dynamic light scattering (DLS) studies were performed on the NP dispersions in DI water with a pH around 7. Results are presented graphically in Figure 5, while they are summarized and compared with the primary particle size obtained from TEM micrographs in Table 2. A polydispersity index (PdI) is also provided for the DLS size estimates, as it reveals the goodness of size distribution where a value less than 0.3 relates to homogeneous population [23]. DLS analysis results for Ru NPs samples showed an average hydrodynamic size varying from 10 to 100 nm, as a result of changing reaction parameters. Ru-5, Ru-2, and Ru-1 samples were prepared at 140, 150 and 160 °C, respectively. Their hydrodynamic size was much larger than their primary NP size, which can be ascribed to the clustering of NPS in the presence of PVP. The cluster size showed variations with the change in reaction temperature, as it strongly depended on the number and size of the NPs held together by the PVP chains. By comparing the hydrodynamic size of Ru-4 and Ru-2, the effect of PVP concentration can be inferred. While the primary particle size was about the same for these samples, the hydrodynamic size was 30 nm for Ru-2 and 70 nm for Ru-4, showing that lowering the PVP content have caused larger clusters. Hydrodynamic sizes of 48 nm for Ru-3 and 30 nm for Ru-2 were obtained when the Ru precursor concentration was halved in the Ru-3 sample. Ru-3 with a smaller primary NP size formed larger agglomerates, probably due to the presence of excessive PVPs holding a great number of small NPs together. As for the effect of the reaction time, samples Ru-2 and Ru-6 showed no big difference in the hydrodynamic radius of agglomerates, despite the difference in the size of primary NPs (Ru-2: 2.5nm; Ru-6: 1.6 nm).

Rh samples showed average hydrodynamic size ranging from 25 to 50 nm. The effect of PVP MW can be seen from samples Rh-1 and Rh-2. A low MW PVP not only led to the formation of smaller NPs, but also formed larger agglomerates. Halving the concentration of PVP from Rh-2 to Rh-3, allowed for the formation of larger agglomerates, in agreement with the case of Ru. Although the temperature of the reaction had a big impact on primary NPs size, seen from Rh-2 (115 °C) and Rh-4 (150 °C), it yielded a slightly larger agglomerate size for Rh-4. Besides the capability of tuning the primary particle size, the conditions studied allow us to obtain NPs with different extents of agglomeration. Rh NPs were observed to have a lower agglomeration extent than Ru NPs. The observed agglomeration of as-made NPs in the neutral pH regime is strongly related to their surface chemistry.

**Figure 6:**
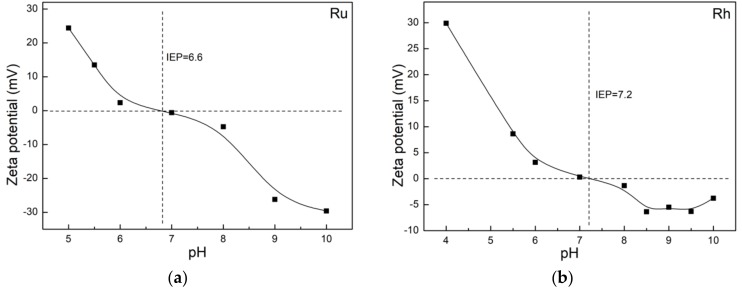
pH vs. ζ-potential of PVP-coated (**a**) Ru (Ru-2) and (**b**) Rh (Rh-2) NPs dispersed in DI water. The pH is tuned by dropwise addition of hydrochloric acid (0.1 M) or sodium hydroxide solution (0.1 M) solution.

The discrepancy between the average particle size estimated from TEM vs DLS (c.f., Table 2) is ascribed to the agglomeration of NPs due to their colloidal stability. Surface charge on the Ru and Rh NP colloids has been studied as a function of pH to evaluate the possible reasons for agglomeration by using Zeta potential (ζ-potential) analysis. The isoelectric point (*IEP*) is defined as the point where the surface charge on NPs is balanced by the attracted, oppositely charged ions, leading to overall zero surface charge. The *IEP* was studied for the as-synthesized Ru / Rh NPs as a function of pH, and the results are presented in Figure 6. Although both the NPs were coated with PVP, they showed slightly different surface charges. The *IEP* for Ru NPs was reached at pH 6.6, while it was at pH 7.2 for Rh NPs. Above these pH values, both NP surfaces showed a weak negative surface charge at the physiological pH of 7.5. The charge mainly originates from PVP [24], thus, the NPs coated with PVP remain dispersed in part by zeta potential and in part due to the steric hindrance created by the large PVP layer coating on their surface. Considering the weak ζ-potential for Ru and Rh NPs dispersion in DI water, this also showed that, generally, smaller primary particles formed larger agglomerates [25,26].

### 3.4. Cytotoxicity Studies

**Figure 7:**
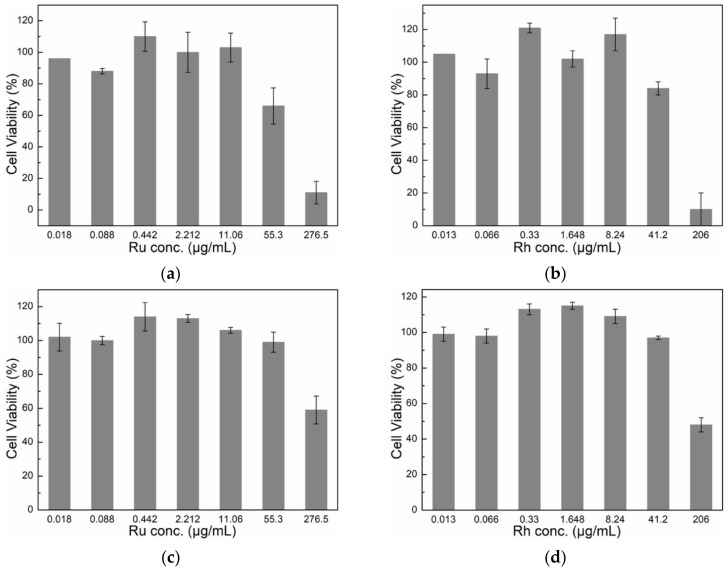
CCK-8 based cytotoxicity assay of Ru and Rh NPs in (**a**,**b**) murine macrophages (RAW 264.3) and (**c**,**d**) human-derived ovarian cancer cells (SKOV-3). The percentage of cell viability is calculated taking negative control cells incubated in the absence of NPs with 100% viability.

The cytotoxicity of Ru and Rh NPs was determined by monitoring the metabolic activity of murine macrophages (RAW264.7) and human ovarian cancer (SKOV-3) cell lines after 24 h exposure to NPs. Ru-2 and Rh-2 NP systems have been chosen due to the fact that, despite having different particle size, they showed a similar hydrodynamic volume, or agglomerate size. Murine macrophages serve as a model for immune cells since macrophages are part of the rapid immune response, important in the removal of foreign material. NPs are generally cleared from the systemic circulation by the mononuclear phagocyte system. As the focus of developing these nanoprobes for XFCT is ultimately cancer diagnostics, the SKOV-3 cell lines were also included to investigate the response of different cell lines to the developed nanoprobes. NP stock solutions were tested for possible LPS contamination [27] using the Endosafe1-PTS™-Assay. The results showed that the LPS values for the stock solutions were below the maximum admissible limit of 0.1 EU/mL [28]. Figure 7 shows cell viability as a function of Ru and Rh concentrations using the CCK-8 assay. It is important to note that the concentration ranges for Ru and Rh NPs were slightly different due to the fact that the serial dilutions were performed from respective stock solutions with different concentrations. We are aware that the highest concentrations exposed are higher than the recommended dosimetry limits [29], though the outcome of the cytotoxicity assay is still valid, as a concentration-dependent response is observed.

**Figure 8:**
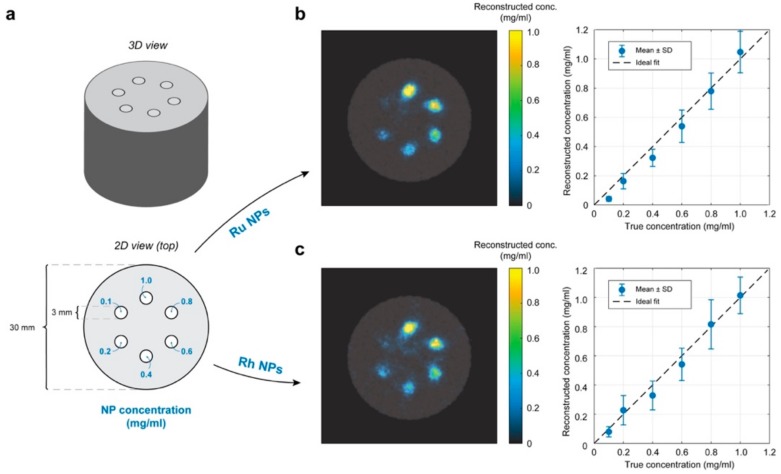
X-Ray fluorescence computed tomography (XFCT) contrast potential of Ru and Rh NPs in a small-animal sized phantom. (**a**) Physical phantom used to mimic a mouse. The phantom body is a 30 mm diameter 3D-printed water-filled cylinder. Cylindrical insets of 3 mm diameter are each filled with different concentrations of Ru and Rh NPs, respectively, for two separate tomographic scans. (**b**) **Left**; Overlay of CT (gray-scale) and XFCT (color) reconstruction of an axial slice through the phantom. The cylindrical insets were filled with Ru NPs at different concentrations. Color bar represents the reconstructed concentration of Ru in the XFCT image. **Right**; Average reconstructed concentration, including standard deviation (SD) in each cylindrical inset plotted against the true concentration. Ideal reconstruction represented with a black dashed line. (**c**) Same as (**b**) but for Rh NPs.

The viability of macrophages (RAW264.7) was significantly influenced by the presence of a high concentration of Ru and Rh NPs when compared with the viability of the SKOV-3 cell line in the CCK-8 assay. Ru NPs influenced the viability of macrophages, reaching about <50% viability at the Ru concentration of 55 μg/mL (Figure 7a). Rh reduced the macrophage viability significantly at the highest concentration of 206 μg/mL, to about 10%, which, upon five-fold dilution, was greatly improved to >80% (Figure 8b). SKOV-3 cell line showed a lower toxicity response in the presence of NPs at their highest concentration, reaching a viability of about 60% for Ru (at 277 μg/mL) and around 50% for Rh (at 206 μg/mL) (Figure 7c,d). This cytotoxicity response in the presence of the NPs might be explained by the different functions that the macrophages and the cancer cells are specialized to perform. While the macrophages should react to any external or internal aggression factors, the cancer cells should adapt to any conditions in order to survive and multiply. We emphasize that the viability reduction is observed in both cell lines only at very high concentrations of μg NPs/mL. However, it is difficult to distinguish the mechanism of the observed viability reduction, or the cytotoxicity, of Ru and Rh NPs from the performed test, as it may be strongly dependent on the morphology and surface chemistry of NPs, among others. The PVP coating is considered bio-compatible. The PVP coating can be detached from the agglomerates in the cell culture media due to active chemical surroundings and a high ionic strength, thus setting free/releasing NPs. Metallic NPs with a very small size have been reported to show cytotoxicity, even for Au, which is otherwise known as biocompatible when >13 nm [30]. We speculate that the observed effect may be simply due to the size of these NPs. The results must be confirmed by further cytotoxicity studies where other factors might be identified.

The biocompatibility of these NPs can be further improved via different strategies, if deemed necessary. One of the common routes, for instance, is the coating of their surfaces with a hard, bio-compatible inorganic shell such as silica, SiO_2_. Colloidal chemical strategies can be integrated into the synthesis process to perform this type of coating for the generation of core-shell structures, where the shell can minimize possible surface-related toxicity problems. We are currently investigating different coating strategies, not only to influence the cytotoxicity but also to use these -OH terminated silica surfaces for the attachment of ligand (peptides, affibodies, and antibodies) for active targeting of tumors.

### 3.5. XFCT Phantom Demonstrations

Figure 8 demonstrates the contrast potential of Ru and Rh NPs for XFCT. Six cylindrical inserts were filled with different concentrations of Ru (Ru-2) and Rh (Rh-2) NPs were dispersed in water, leading to a metallic content of 1.0, 0.8, 0.6, 0.4, 0.2 and 0.1 mg/mL (see Figure 8). The concentrations of Ru and Rh in the stock solutions were determined as 2.765 mg/mL (2765 ppm; 2765 μg/mL) and 2.060 mg/mL (2060 ppm; 2060 μg/mL) respectively, using the ICP-OES method, prior to the XFCT experiments. Apart from the lowest concentration, the cylindrical insets can be clearly distinguished from the rest of the phantom in the reconstructed images (c.f., Figure 8(b,c,left)). The reconstructed concentrations for both Ru and Rh NPs display an expected linear relationship with the true concentrations (c.f., Figure 8(b,c,right)). From the images, we can infer that concentrations as low as ~0.2 mg/mL (200 μg/mL) of both Rh and Ru NPs can be reliably separated from the background noise present in the images. For comparison, in our recent small-animal XFCT study with Mo-based NPs we observed down to 0.5 mg/mL (500 μg g/mL) Mo in the large organs after 24 h [1]. The background noise arises due to the detection of Compton scattered incident X-rays that overlap in energy with the characteristic Kα X-Ray fluorescence of the NPs. The background contribution has been estimated and removed in the XFCT reconstruction, however, any attempt to separate XRF from overlapping background is imperfect. It is well-known that the magnitude of the background signal detected at the XRF peak is a critical factor limiting the sensitivity in XFCT. This explains why background noise is more present in the Rh XFCT reconstruction as compared to the one for Ru (c.f., XFCT spectra in Supplementary File Figure S2).

## 4. Conclusions

XFCT, as an emerging biomedical imaging technique, urges the need to develop new probes/contrast agents, with X-Ray absorption edge energies matched to the X-Ray excitation energy for optimal signal generation. For this purpose, Ru and Rh NPs were synthesized by the polyol method using ethylene glycol, in the presence of PVP as a stabilizer, with varying molecular weights, and at different reaction focusing temperatures. Resultant materials were characterized in detail using a library of techniques including XRD, TEM, FT-IR, DLS, zeta potential and TGA. Their crystallinity was confirmed by XRD, while morphology and particle size were evaluated by TEM, revealing individual spherical Ru particles in the size range of 1–3 nm, and triangular Rh particles of 6–9 nm. A surface coating polymer layer was evidenced by FT-IR and TGA analyses, while the colloidal stability was demonstrated by the weak negative charge around the physiological pH. When dispersed in physiological pH, both material systems showed an agglomeration of formed particles into larger assemblies with the range of sizes 12–104 nm for Ru and 25–50 nm for Rh, due to the presence of a PVP layer holding them together. Two cell lines, macrophages (RAW264.7) and ovarian cancer (SKOV-3), were used for the evaluation of cytotoxicity of selected Ru and Rh NPs. A significant viability reduction was seen for macrophages at the highest NP dose, while the viability was still at 50% and above for the SKOV-3 cell line. XFCT performance was evaluated on a small-animal-sized phantom, showing a promising quantitative evaluation of the NP concentrations with an expected linear response. The lowest visible NP concentrations agreed with what has been observed in our previous small-animal XFCT experiments [1], indicating the potential capacity of the presented Rh and Ru NPs as XFCT contrast agents in small-animals. From a contrast perspective, the metallic Ru and Rh NPs suffer from higher background noise compared to those based on Y, Zr, Nb and Mo oxide NPs, reported earlier [1,4]. However, the metallic NPs have benefits that include a smaller size (<10 nm) and facilitated surface functionalization. This work provides a detailed route for the synthesis, size control and characterization of two materials systems as viable contrast agents for XFCT applications. The biocompatibility of these NPs can be further improved for high dose in-vivo administeration for eventual bio-imaging applications.

## Figures and Tables

**Table 1:** Synthesis conditions for Ru and Rh NPs by polyol method: amount of precursor, reaction temperature, reaction time, amount and molecular weight of PVP are specified for each sample.

Sample	Precursor (mmol)	T (°C)	Reaction time (h)	PVP (mol) [MW] (kDa)
RuCl3·xH2O				
Ru-1	0.2	160	1.5	0.004 [10]
Ru-2	0.2	150	1.5	0.004 [55]
Ru-3	0.1	150	1.5	0.004 [55]
Ru-4	0.2	150	1.5	0.002 [55]
Ru-5	0.2	140	1.5	0.004 [55]
Ru-6	0.2	150	0.5	0.004 [55]
RhCl3·xH2O				
Rh-1	0.2	115	1.5	0.004 [10]
Rh-2	0.2	115	1.5	0.004 [55]
Rh-3	0.2	115	1.5	0.002 [55]
Rh-4	0.2	150	1.5	0.004 [55]

**Table 2:** Particle size estimations for as-synthesized Ru and Rh NPs according to Table 1. Average particle sizes obtained by counting particles from TEM micrographs, and by DLS measurements reported in hydrodynamic volume %; polydispersity index (PdI) is also provided for the DLS measurements.

Sample	Particle Size, TEM (nm)	Particle Size (Volume)D-Average (nm)—[PdI]
Ru-1	1.61	104.2—[0.108]
Ru-2	2.51	29.86—[0.247]
Ru-3	<1	47.86—[0.206]
Ru-4	2.52	69.73—[0.097]
Ru-5	~1.5	12.23—[0.211]
Ru-6	1.58	33.42—[0.226]
Rh-1	7.02	47.80—[0.271]
Rh-2	6.06	25.71—[0.301]
Rh-3	6.40	35.13—[0.214]
Rh-4	8.74	29.09—[0.228]

## Supplementary Materials

The following are available online at https://www.mdpi.com/2079-4991/10/2/310/s1, Figure S1: XFCT spectra for Ru and Rh NPs, Figure S2: Thermogravimetric analysis (TGA) of as synthesized PVP-coated (**a**) Ru (Ru-2) and (**b**) Rh (Rh-2) NPs.
