# Supplementary material for: Synthesis and Cytotoxicity Studies on Ru and Rh Nanoparticles as Potential X-Ray Fluorescence Computed Tomography (XFCT) Contrast Agents
Source: Nanomaterials (Basel). 2020 Feb 12;10(2):310. doi: 10.3390/nano10020310 (PMC7075218; doi:10.3390/nano10020310)
Supplement: Supplementary file 1 [file nanomaterials-10-00310-s001.pdf]

Electronic Supplementary Material
for

Synthesis and cytotoxicity studies on Ru and Rh nanoparticles as potential X-ray fluorescence computed tomography (XFCT) contrast agents

Yuyang Li, Kian Shaker, Martin Svenda, Carmen Vogt, Hans M. Hertz, Muhammet S. Toprak*

Biomedical and X-Ray Physics, Department of Applied Physics, KTH Royal Institute of Technology, Stockholm, Sweden

* Correspondence: toprak@kth.se (MST); Tel.: +46-735-519358

Figure S1 shows the TGA thermograms, alongside differential thermograms (DTG), of dried powders from as-made Ru-2 and Rh-2 NP samples. For both the samples, there is a significant amount of polymer/PVP on the NP surfaces, which decomposes in the temperature range from around 200 °C to 500 °C with a corresponding weight loss. This loss is more pronounced in the DTG thermogram centered around 400 °C, reaching almost 44% by weight for Ru NPs and 57% by weight for Rh NPs. Due to the very small size of NPs, and the large surface area to volume ratio, there is enormous surface which is coated/passivated by the polymer chains. This is favorable as PVP is a water-soluble nonionic polymer with the hydrophilic carbonyl groups, and is expected to stabilize the NPs.

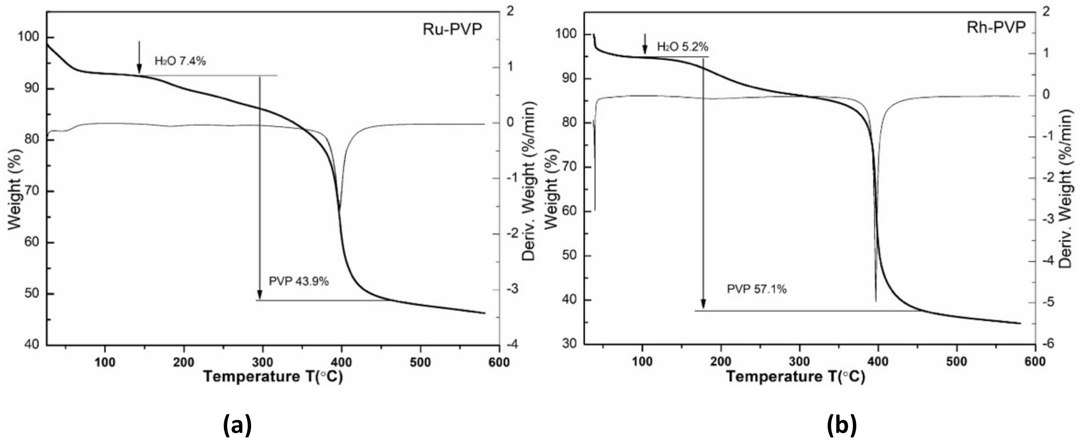

Figure S1. Thermogravimetric analysis (TGA) of as synthesized PVP coated (a) Ru (Ru-2) and (b) Rh (Rh-2) NPs. For sample details see **Table 1**.

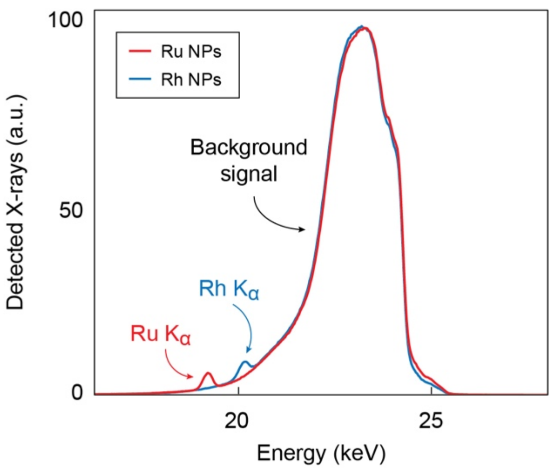

Figure S2 | XFCT spectra for Ru and Rh NPs. Detected XFCT spectra, for improved statistics integrated over the whole phantom scan with Ru (red) and Rh (blue) NPs. The K_α peaks of Ru (19.26 keV) and Rh (20.21 keV) are clearly visible. The background level at the Rh K_α peak is higher than that at the Ru K_α peak, explaining the differences in background noise seen in the reconstructed XFCT images (*c.f.*, **Figure 8**). Note that detected spectra per pixel (not shown) is significantly noisier making it more challenging to separate background from the x-ray fluorescence signal.
